# Sexual behaviour, human papillomavirus and its vaccine: a qualitative study of adolescents and parents in Andalusia

**DOI:** 10.1186/s12889-021-11510-4

**Published:** 2021-07-28

**Authors:** María González-Cano, Francisco Garrido-Peña, Eugenia Gil-Garcia, Marta Lima-Serrano, María Dolores Cano-Caballero

**Affiliations:** 1 Department of Nursing, University of Seville, Seville, Spain; 2 Department of Criminal Law, Philosophy of Law, Moral Philosophy and Philosophy, University of Jaen, Jaen, Spain; 3 Department of Nursing, University of Granada, Granada, Spain; 4 Biosanitary Research Institute of Granada, Granada, Spain; 5 University Hospital Virgen de las Nieves, Granada, Spain

**Keywords:** Parents, Adolescents, Sexual behaviour, Papillomavirus vaccine, Human papillomavirus

## Abstract

**Background:**

Human papillomavirus (HPV) is one of the most common sexually transmitted infections and can be prevented by vaccination. The purpose of this study is to gain a better understanding, by analysing interview responses of adolescents and parents, of how adolescent sexual behaviour is approached in families, how widespread knowledge about HPV is in Andalusia, the autonomous region with the lowest vaccination rate in Spain, as well as to learn more about the interviewees’ position regarding vaccination.

**Methods:**

A qualitative study by means of 15 focus groups of adolescents (*N* = 137, aged 14–17 years) and five focus groups of parents with children of those ages (*N* = 37) was conducted in the provinces of Granada, Seville and Jaén (Andalusia, Spain). The audio data were transcribed verbatim, coded and analysed thematically using NVIVO-10 software.

**Results:**

There were three major results: (1) There is a lack of communication between adolescents and parents regarding sexual behaviour; (2) In both groups, scarce knowledge about HPV and vaccination was found; (3) Parents mistrust vaccination due to a lack of qualified and verified information about its benefits.

**Conclusions:**

Healthy adolescent sexual behaviour is aided by communication within the family. Families need more information based on the evidence about HPV and vaccination. Health professionals are a key element in this process.

**Supplementary Information:**

The online version contains supplementary material available at 10.1186/s12889-021-11510-4.

## Background

Adolescence is a key period for the acquisition and consolidation of a lifestyle. During this period, both healthy and harmful habits are established and reinforced [1]. The need for acceptance by others and a low perception of danger can lead adolescents toward risky health behaviours [2].

The prevention of risky behaviour must be approached from different spheres, among which the family stands out [3]. For children, parents serve as a model for the expression of emotions, gender role, sexuality and human relationships [4]. Strong family bonds can help children maintain similar relations of intimacy and affection with their peers [5]. When sexuality and sexual behaviour is not addressed openly in the family, children might perceive that it is not appropriate to talk about the subject [4]. Some authors point out that family environments with poor communication and/or where the adolescent does not receive sufficient support, can lead to a higher probability of the adolescent engaging in risky sexual behaviour, such as not using a condom and/or starting sexual relations at an early age [5]. Due to the combination of a reluctance in adolescents to talk about sexuality with their parents, and a possible lack of knowledge in some parents on how to address this issue with their children, the collaboration of expert professionals is often necessary [4]. Adolescence is a stage in which youngsters face numerous challenges [6], including their identity, sexual self-image and sexuality. They often feel insecure, need the acceptance of the group and are easily influenced by their peers’ opinions [4].

In Spain, the age for first sexual intercourse in girls and boys has decreased in recent decades [6, 7]. The interval in which adolescents are sexually active has been extended; the age of first sexual intercourse has decreased from 17.5 for young adults who are now 22–25 years old to 15.5 for adolescents who are now 16–18 [7]. In addition, a considerable decrease in adolescents’ concerns about sexually transmitted infections (STI) might lead to an increase of STIs, especially among the youngest [6, 7].

Human papillomavirus (HPV) is one of the most common STIs. It is estimated that between 80 and 90% of people who have sexual relations have been or will be in contact with the virus [8]. The prevalence of HPV infection in normal cytology worldwide is estimated to be between 11 and 12%, being the highest for females below 25 years of age [9].

HPV infection can lead to a variety of consequences, including cervical cancer. In Europe, this type of cancer is the second most frequent in females aged between 15 and 44 years [9]. It is estimated that 61,072 new cases arise each year in Europe, leading to around 25,829 deaths per year as a direct consequence. In Spain, 1942 cases of cervical cancer are diagnosed every year, causing 825 deaths per year [10].

Moreover, the infection can lead to other less known but very important consequences, such as genital warts and oropharyngeal, penile, anal, vulvar and vaginal cancers [10]. Some types of cancer caused by HPV show a higher prevalence in males than females. Data from the United States show that the prevalence of high-risk oral HPV infection in the general population is higher in males than females (7.3 and 1.4%, respectively) [11].

Currently, evidence shows that the HPV vaccine is successful in reducing the prevalence of HPV infection and its consequences [12]. In Spain, three vaccines are marketed, classified according to the genotypes that they contain: bivalent (16, 18), quadrivalent (6, 11, 16, 18) and nonavalent (6, 11, 16, 18, 31, 33, 45, 52, 58) [13]. Since 2007, HPV vaccination has been included in the Spanish national public health system and is freely available for females from 12 years of age [14]. The administration of HPV vaccines varies between autonomous communities, with some regions choosing school-based and others clinic-based administration. In Andalusia, vaccines are administered in primary health centres [14].

In 2018, the uptake rate in Spain of the first and second dose of the HPV vaccine was 84.9 and 72.8% respectively [15]. Considering those figures, Andalusia is the autonomous community with the lowest HPV vaccine uptake rates in Spain: 75.2% for the first dose and 59.9% for the second [15]. Although little data exist on overall uptake rates in Europe, Spain ranks among the countries with the highest vaccination rate when compared to other countries with available data [13].

The qualitative approach to HPV vaccination from the perspective of adolescent girls and their parents has been studied by different authors, focusing on the reasons that lead to accepting or refusing the vaccine. These investigations mention as some of the reasons that influence vaccine refusal, a lack of information from parents about the risks and consequences of HPV infection, fear of vaccine safety as well as doubts about its efficacy, and the belief that daughters were too young to be sexually active [16–23]. According to these studies, parents were more motivated to have their children vaccinated when the recommendation came from an authority, when they received advice from their health professionals, and when they had a strong desire to prevent illness in their daughters [16, 18, 19, 24].

There are few studies that analyse a social discourse around HPV and vaccination in Spain, especially by adolescents [25]. Our research approaches this topic from the perspective of its protagonists, emphasising the study of the specific factors at work in the region of Andalusia, which has the lowest HPV vaccination uptake rates in Spain [15].

In view of these circumstances, the aim of this study was to identify how adolescent sexual behaviour is approached in families by analysing interview answers from adolescents and parents, as well as to explore their knowledge about HPV and learn more about their position regarding vaccination.

## Methods

A study was carried out using a qualitative methodology with a content analysis about adolescent sexual behaviour, HPV and its vaccine with adolescents and parents to learn more about the interviewees’ experiences and opinions. This study involved 20 focus groups (FGs) and was carried out between February and May 2017 in the cities of Jaén, Granada and Seville (Andalusia, Spain).

### Sample

The participants were students aged between 14 and 17, and parents with children of those ages. This age group coincides with the average age range for the first sexual intercourse [7] and allows for previous HPV vaccination. The participants were contacted through their school because, since education is compulsory at that age range, adolescents spend most of their time at school.

A purposive sample was used. First, we got in touch with primary care nurses assigned to the schools who helped us communicate with the educational institution. After receiving agreement from the schools, headmasters and school counsellors reached out to the families and agreed on a date to conduct the FGs. Prior to the creation of a FG, informed consent was obtained from parents and adolescents.

Fifteen FGs with students were created, with a total of 137 participants. Student FGs were created in six state-subsidised schools, two private schools and seven public schools.

Five parent FGs were created, with 37 participants in total, in two state-subsidised schools and three public schools. The inclusion criterion was to have children of the ages described above.

The profile of the students and parents who participated is shown in Table 1.

**Table 1:** Characteristics of participants in focus groups

Variable		N adolescents (137)	N parents (37)
**Age (mean, SD)**		15.12 (±0.75)	47.45 (±3.82)
**Sex**	Female	82 (59.85%)	35 (94.6%)
	Male	55 (40.15%)	2 (5.4%)
**HPV vaccination**	Yes	61 (74.4%)	
	No	16 (19.5%)	
	Unkown	5 (6.1%)	
**Education level**	Elementary		7 (18.9%)
	GCSE^a^		5 (13.5%)
	GCE^b^		3 (8.1%)
	Vocational training		11 (29.7%)
	University		11 (29.7%)

^a^GCSE: General Certificate of Secondary Education

^b^GCE: General Certificate of Education

### Data collection

In order to obtain the information from each FG, an ad hoc script was used (supplementary material), maintaining the same categories of the script for both groups and modifying the wording of the questions to adapt them to each group.

Before the start of an interview, researchers were introduced to participants and the goals of the study were clearly explained. All interviews were conducted in a classroom assigned by the school headmaster, making sure each interview could take place in a quiet and accessible environment, with adequate temperature and light conditions [26].

The average duration for the compilation of information was 40 min per group. All sessions were recorded using two separate digital recorders, with participants’ permission, and notes were taken in order to facilitate transcription.

A specialist paediatric nurse, with background in the phenomenon under study and qualitative research, moderated each FG session. A nurse and anthropologist with training in qualitative research, observed the session, took notes and recorded audio. The groups were identified with the letters FG plus the letter ‘A’, in the case of the adolescents and the letter ‘P’ in the case of the parents, followed by the number of the group. In order to maintain their anonymity, each group member was also given a number according to their order of participation.

### Analysis

A summative content analysis was carried out as proposed by Hsieh and Shannon [27]. This type of analysis combines the creation of categories through two approaches, deductive category application and inductive category formation. Firstly, through the deductive approach, the main categories were created, derived from the research question and the theoretical frame of reference. Subsequently, emergent categories were identified arising from the detailed and repeated reading of the transcribed texts.

In order to guarantee rigor of our methodology and to enhance trustworthiness [28], two researchers independently coded the transcripts. Finally, the categories, their definitions and the texts assigned to each of these categories were triangulated with the other team members. For codification, the qualitative analysis QSR NVivo 10 software was used.

#### Ethics approval

All participants signed a consent form, where they were informed of the purpose and objectives of the research and their participation. In the case of students, parents signed a consent form. They were also informed of their right to abandon the investigation at any time without any consequence. The confidentiality of the participants’ data was ensured during the entire investigation process. Prior to beginning the research, the approval of Granada Provincial Research Ethics Committee was obtained. This committee is integrated in the Network of Ethics Committees of the Andalusian Public Health System.

## Results

### The approach to adolescent sexual behaviour in the family

The adolescents express that they do not approach that topic with their parents because they are ashamed and when they do, the conversation almost always tends to revolve around the use of contraceptive methods.

*‘Whenever we talk about it, the little that I talk about it with them, I’m told to do it with protection and caution and to make sure it’s with someone who won’t spill the beans’* (FGA2-2)

*‘It’s an embarrassing topic for us’* (FGA1-1)

They consider that their parents have different recommendations about sexual behaviour and that these depend on sex. Boys and girls’ parents insisted on the importance of protection in both, but besides, parents oriented girls to have sex with a definitive partner. Additionally, they receive more messages aimed at self-control in sexual behaviour.

*‘I’m told to do it once I have my definite boyfriend, when it’s the right moment’* (FGA11-8)

The adolescent girls relate that sexual practices of boys and girls are assessed socially in different ways: having initiated sexual relations is added value for boys and a dishonour for girls.

*‘If we were boys maybe it’d be easier for us, because if you are and lose your virginity very young, you rock, but if you’re a girl, you become a streetwalker’* (FGA13-3)

*‘If a girl does it at 15 or 16 years old, she gets a bad reputation’* (FGA15-5)

Regarding risky sexual practices, many express that they know peers who perform them, such as not using condoms or having multiple sexual partners at early ages.

*‘I know people who have done it unprotected and take the pill to avoid pregnancy, she takes the morning-after pill and being younger than me; I’m 15, so imagine. At 13, 14 … ’* (FGA11-3)

*‘After 14 almost everyone (has had sexual relations), the vast majority’* (FGA12-6)

Opposite to these statements are those of the parents who participated; they justify not addressing issues of sexual behaviour with their children because they are not interested in that topic and they are too young to engage in sexual relations.

‘*Personally, I really don’t understand; first because they’re young, they don’t have a partner’* (FGP4-2)

*‘They’re 14, they’re too young’* (FGP5-7)

*‘I think he is more entertained with the computer’* (FGP5-6)

### HPV

Regarding their knowledge about HPV, parents and adolescents think that they have scant knowledge and whenever they received any kind of information, it has been related to vaccination.

*‘When we were given the vaccine, we were given a small introduction’* (FGA6-2)

*‘For me, it’s a virus I know little about, only when I had my daughter vaccinated, but I don’t have much idea’* (FGP2-3)

Focusing on the transmission mode and means of prevention, different levels of knowledge are demonstrated by parents and adolescents– while the former state that it is sexually transmitted and consider a condom to be protection from contagion, adolescents consider that it is transmitted by fluids passing from the male to the female, even by lack of hygiene.

*‘I’ve been told and I’ve read it’s when they have sex, it’s transmitted by having sexual relations, so it’s important that they use a condom’* (FGP1-7)

*‘By semen’* (FGA12-3)

*‘By blood, kissing, anything’* (FGA9-6)

*‘I think it can also be transmitted by lack of hygiene, by fungus or something’* (FGA9-5)

In general, both students and parents consider that males are not affected by the infection and that if this was the case, the consequences would be less than for females.

*‘Boys don’t, I think they can only pass it on’* (FGA11-4)

*‘They can have it, but not as much as women’* (FGA3-8)

*‘It seems that vaccinating women and men is because we girls suffer more than them, consequences are for us girls, not as much for boys’* (FGP5-6)

### Vaccines

Adolescents do not have an informed opinion about the use of vaccines, they accept the decision that their parents make in this respect. Coinciding with this, none of the parents expressed that they talk about this topic with their children or ask them about it.

*‘My father agrees with the vaccine, my mother always tells me to get the vaccine when they come to give it’* (FGA14-2)

*‘Her father and I are the ones who decide on his vaccination, it is not something that is discussed at home’* (FGP1-1)

In general, parents trust vaccines and admit their utility, although sometimes they question them due to a lack of transparency and the existence of lucrative reasons related to the economic interests of the pharmaceutical industry.

*‘I question vaccines because I believe there’s little clarity. It’s not that I consider them useless, I see there are many lucrative things around vaccines and pharmaceutical companies and sometimes they confuse us on purpose’. ‘It seems to me that if it’s obligatory, it should be in the vaccination schedule and force the social security to finance it’* (FGP2-5)

### HPV vaccine

The adolescents who participated have scant knowledge about the HPV vaccine and sometimes this knowledge is contradictory. In their statements, they claim that it produces cancer or that it is dangerous, based on comments that they have heard.

*‘A friend of mine went to the doctor and asked about that vaccine and they said it may cause cancer or whatever, so my mother went with my friend’s mother, heard that and said I wouldn’t get it’ (*FGA15-1)

Similarly, the parents express that they have scant information about the convenience of vaccinating their daughters. When some participants asked professionals about vaccinating their daughters, it was suggested to do what they feel convenient at their own risk, giving a feeling of uncertainty regarding whether they are doing the right thing.

*‘I asked too … the nurse asked me if I wanted her to be vaccinated, or I didn’t want, but she told me it was there for it, but said “well, I leave it to you” Right, the responsibility is mine, but tell me if it’s good give her the vaccine; but if you haven’t said yes or no, then I don’t know what to do´* (FGP4-1)

*‘In my case, no information at all. You went to your appointment, can I help you?, papillomavirus, age, jab, immunisation record, stamp and go home’* (FGP1-1)

Another aspect mentioned in the statements was the relation between the vaccine and sexual relations. In both groups, we found a belief that once they engage in sexual relations, the vaccine cannot be administered because it is not efficacious.

*‘I’ve been told that you can’t have sexual relations before getting vaccine because it doesn’t work’* (FGA15-3)

*‘One of the things the nurse told my daughter when she went to get the vaccine was: “now you can’t have sexual relations”’* (FGP5-3)

When approaching the vaccine, an important aspect is the effects after vaccination. In the case of adolescent girls, none had suffered adverse effects although we found the testimony of two girls who related that a relative had suffered a reaction after being given the vaccine, although the relation between both episodes was not confirmed.

*‘My cousin still hasn’t been diagnosed but got lumps and many things after getting the second dose of the vaccine as adverse effects; she wasn’t given the third dose for that reason. But it hasn’t been confirmed that it’s because of the vaccine’ (*FGA11-1)

In the case of the parents, all but one mother related that their daughters had not suffered any adverse effect. This mother commented that they still had not been able to clearly establish the relation, that it was she who had established her daughter’s health issues as a consequence of administration of the vaccine.

*‘She got it at school. Three days later, she started with huge otitis. She got antibiotics, later she got pharyngitis, after that sinusitis, she felt terrible. I wondered “is it related to the vaccine? No, how can it be? It’s a coincidence, that’s all” … Since then, she started not getting her period … Huge headaches … To sum up, it’s been 5 years now and this summer she has presented tremors … We don’t know why; I think it may still be a reaction to the vaccine’* (FGP3-2)

## Discussion

In our research, we found that adolescents do not want to talk about sexual behaviour in their homes and that when a conversation takes place, it mainly revolves around contraceptive methods. Regarding HPV, the knowledge of both groups is scant. Focusing on vaccination, we find the same situation, a lack of information in both groups and complaints from parents about the lack of information clarity provided by health professionals in this regard.

Sexual education comprises both informal (e.g. through family) and formal education (e.g. through schools). The results of our research coincide with other studies regarding the approach to informal sexual education in families, where parents and adolescents talk very little about sexual behaviour, being for adolescents their peers the main source of sexual information [29, 30]. Regarding formal education, although some adolescents consider talks in schools as the most useful source of information on sexuality and sexual behaviour [6], in Spain, sexuality education is not regulated. Whether it is taught or not depends on each school and sexuality-education workshops are usually implemented by external organisations [31]. In Andalusia, there is a specific programme to approach health promotion in schools called the ‘Forma Jóven’ programme, which is taught by health professionals, but this strategy is sometimes not sufficient since the professionals themselves note that education is started too late and little time is dedicated to it [32].

We found gender differences which coincide with the findings of another study, where social double standards are proven to exist: it is accepted with greater complacency that boys engage in sexual relations in comparison with girls [33]. Even on some occasions, parents advise girls to avoid relations to a greater extent than they advise boys [6]. In our research, adolescents consider that sexually active girls are more socially frowned upon than boys. These statements are supported by other studies where the participants consider that boys are more interested in sex than girls [6] and affirm that sexually active females have less worth and that the active and seductive role corresponds uniquely to males [34].

Students of FGs were between 14 and 17 years old, which coincides with the beginning of sexual activity according to various studies [7, 35], and as also noted by the participants. This contrast with the statements of mothers who claim that their children are too young to engage in sexual relations. This thought may be considered a barrier when it comes to communication [36]. It is important to make parents aware of the fact that sexuality is part of their children’s lives and is something positive which they are going to face. We consider that a strategy which may help is for parents to participate in the training about sexuality and sexual behaviour which is given to adolescents [37].

Interesting similarities and differences between parents and students emerged regarding the knowledge about HPV and its vaccine. In the discourse, as similarity of both groups, we find the general scarce of knowledge that coinciding with other studies [38]. As the main difference, while parents know the transmission mode, students relate it to fluids [39] or lack of hygiene [40]. Due to the lack of knowledge about this infection, they may fill the knowledge gap with what they know about other STIs. This leads to the fact that in the verbatims of parents and students we find that the risk of infection is underestimated, especially in men, which coincides with previous studies [41].

The gender bias found in both groups is striking since females are more penalised for their sexual practices by considering that the infection in males has less severe consequences [38]. This may lead individuals to think that males are less at risk and can have relations more freely, with the responsibility for protection falling onto females. The lack of awareness of the consequences of the infection in males may be reinforced by the fact that in the vaccination schedule in Spain, the HPV vaccine is only included for females. The vaccination of males against HPV has proven to be an efficacious measure for a greater reduction in the incidence of the virus [42].

Regarding vaccination, parents are mostly in favour. This is reflected in the vaccination rates in Spain, which are around 97% for the primary vaccination and 95% for the booster [15]. One of the most frequent reasons for rejection of vaccination is the belief that economic interests of pharmaceutical industries prevail over the protection of health [43, 44]. Both groups of participants agree that the parents have not talked with their daughters and sons about the vaccines. As Galbraith-Gyan et al. points out [45], not considering the concerns of adolescents on the subject can be a limitation for them, since in the future they will be responsible for their health care decisions. In the case of the HPV vaccine, in the verbatims of parents and adolescents, we find similarities concerning the lack of knowledge. Parents’ doubts regarding the HPV vaccine come from a lack of information given by healthcare professionals, which leads to questioning the effectiveness and importance of the vaccine [22, 46]. As we have shown in our research, the knowledge of HPV that the participants have is related to the vaccine, so those adolescents who had received the vaccine and their parents had a little more information than the rest. If the information about the virus and its vaccine had reached more adolescents, perhaps the uptake rates would have been higher in this population, as pointed out by Camaño-Puig et al. [17]. Some authors also suggest as a barrier to vaccination that parents think that their children are not sexually active yet [23]. In our study we have not found a relationship in the discourses of parents and adolescents about HPV vaccination and sexual behaviour, although it is true that one of the existing myths about the vaccine is the fact that it increases promiscuity, different investigations have shown that is not true [47, 48]. It is important that professionals are informed in order to transmit reliable information based on evidence to parents. On some occasions, the lack of knowledge of professionals leads them to present unclear statements concerning the HPV vaccine and to also have doubts about it [32]. Evidence suggests that the vaccine is ideal for administration at early ages, but we found recommendations from scientific societies which claim that sexually active females may benefit from its protective effects [49]. In addition to the above, in Andalusia in particular, the vaccine is administered in primary health centres instead of schools. This together whit the lack of knowledge and information about HPV and its vaccine, could be the reason for the lower rates of HPV vaccine uptake. A previous study showed in the regions of Spain that HPV vaccine was administrated in schools-based programs the HPV vaccine uptake rate was 14% higher than in the autonomous communities that administrated it in primary health centres [50]. This also concurs with previous investigations that showed that HPV vaccine uptake rate is higher in those countries with school-based programs [48, 51, 52].

In general, the participants informed of no cases of adverse effects due to the vaccine except for three participants, two in the group of students and one informed by a mother who related that a relative had suffered a reaction after being given the vaccine. Several studies collected by the Global Advisory Committee on Vaccine Safety of the World Health Organization show that there is no causal relation between the HPV vaccine and the emergence of illnesses such as Multiple Sclerosis and other autoimmune illnesses [53]. Other research, published recently, shows that vaccine use is not associated with an increased risk of autoimmune disorders, although this poses the necessity of conducting more studies in males in order to know the effects of the vaccine on them [54]. A revision of studies, which included 57,580 people, claimed that the vaccine had an acceptable benefit/risk profile [55]. In a revision carried out by Arbyn et al., the vaccine was proven to be safe, with no differences between groups which received the vaccine and those who received a placebo regarding major and minor systemic adverse effects [56]. However, it is worth mentioning that among the criticisms of several aspects that this revision received is the issue of severe adverse effects [57].

### Strengths and limitations

A strength of this study is that both parents and adolescents’ perceptions on sexual behaviour, HPV and vaccination are included, allowing a comparison of differences and similarities in the views of both groups. There were advantages to discussing these topics in a group setting since participants gave one another mutual support and individuals could choose when to speak. The interviewers introduced themselves as nurses; this contributed to a better willingness of the participants to share their points of view during the interviews and ask questions at the end of the FGs. In addition, this study includes both adolescent boys and girls, which has allowed us to identify sex differences in opinions regarding HPV infection and its vaccine. Furthermore, the parent discussion group includes mothers of both boys and girls, which has enabled us to specifically analyse families of adolescents of both sexes.

The fact of not being able to count on more opinions of parents and not having done FGs with them in private schools could be considered a limitation, since their arguments could have contributed another perspective in the groups. We also want to highlight the fact that the convening of the parents’ meeting was made by the school, which meant that the parents with less time availability did not attend, so their argument is absent. In order to maintain anonymity, the relationship between parents and children was not taken into account; this limitation prevents a broader analysis of the results. Our study reflects perceptions in a specific context and environment, which in our case is the adolescent from Andalusia that attends school regularly. More research is needed to elicit views in different environments.

## Conclusions

It is necessary for families to approach children’s sexual behaviour as part of their education, without ignoring it, without mistrust and with a gender perspective. Families need more scientific information based on evidence about the consequences of HPV infection as well as the benefits and effects of vaccination. Healthcare professionals (physicians and nurses) are a key element for families when making decisions about the HPV vaccine. The information they provide influences the health care quality offered and this must contribute to informed decision making.

## Supplementary Information

**Additional file 1.** Table S1. Categories and script of questions for focus groups.

## Data Availability

The datasets used and/or analysed during the current study are available from the corresponding author on reasonable request.

## Abbreviations

HPV: Human papillomavirus
STIs: Sexually transmitted infections
FGs: Focus groups
GCSE: General Certificate of Secondary Education
GCE: General Certificate of Education
