# Supplementary material for: Sexual behaviour, human papillomavirus and its vaccine: a qualitative study of adolescents and parents in Andalusia
Source: BMC Public Health. 2021 Jul 28;21:1476. doi: 10.1186/s12889-021-11510-4 (PMC8320038; doi:10.1186/s12889-021-11510-4)
Supplement: Supplementary file 1 — Additional file 1. Table S1. Categories and script of questions for focus groups. [file 12889_2021_11510_MOESM1_ESM.docx]

| **Categories** | **Questions for adolescents** | **Questions for parents** |
| --- | --- | --- |
| **Sexual behaviour** | - Do you talk about sexual behaviour with your parents? Which topics do you discuss with them? Do they give you recommendations? - If you have questions about sexual behaviour, who do you ask about? - Do adolescents of your age have sexual intercourse? | - Opinions about the sexual behaviour of your children - Do you talk about sexual behaviour at home? Who do you talk with? Which topics do you discuss? When? |
| **HPV** | - Are you aware of the symptoms of HPV? How is it transmitted? - Who does it affect? - What are the consequences of HPV infection? - How do you know if you are infected? - How do you prevent its contagion? What is the treatment after infection? | - Do you know the symptoms of HPV? How is it transmitted? - Who does it affect? - What are the consequences of HPV infection? |
| **Vaccines** | - What is your opinion about vaccines? | - What is your opinion about vaccines in general? And your partner’s, what is their opinion? - Are your children vaccinated? |
| **HPV vaccines** | - Are you vaccinated at school? - Have you been informed about HPV vaccines? - What is the purpose of the vaccine? - Who must be vaccinated? - When is the vaccine given? What is the age limit to have it given? - Can you be vaccinated if you have had sexual relations? - Have you had adverse effects after the vaccine or know anyone who has? | - Have you been given information about the vaccine? - Have you asked or searched information about it? Where? - Who do you ask for information? - What is the purpose of the vaccine? - Who must be vaccinated? - Can they be vaccinated if they have had sexual relations? - Has your daughter or someone that you know had adverse effects after getting the vaccine? |

**Supplementary file 1**

**Table S1.** Categories and script of questions for focus groups.
